# Concomitant preoperative airflow obstruction confers worse prognosis after trans-thoracic surgery for esophageal cancer

**DOI:** 10.3389/fsurg.2022.966340

**Published:** 2023-01-16

**Authors:** Ke Lang, Xiaocen Wang, Tingting Wei, Zhaolin Gu, Yansha Song, Dong Yang, Hao Wang

**Affiliations:** ^1^ Department of Pulmonary and Critical Care Medicine, Zhongshan Hospital, Fudan University, Shanghai, China; ^2^ Shanghai Key Laboratory of Lung Inflammation and Injury, Shanghai, China; ^3^ Department of Thoracic Surgery, Zhongshan Hospital, Fudan University, Shanghai, China

**Keywords:** esophageal cancer, survival, airflow obstruction, lung function, decision-making

## Abstract

**Background:**

Airflow obstruction is a critical element of chronic airway diseases. This study aimed to evaluate the impact of preoperative airflow obstruction on the prognosis of patients following surgery for esophageal carcinoma.

**Methods:**

A total of 821 esophageal cancer patients were included and classified into two groups based on whether or not they had preoperative airflow obstruction. Airflow obstruction was defined as a forced expiration volume in the first second (FEV_1_)/forced vital capacity (FVC) ratio below the lower limit of normal (LLN). A retrospective analysis of the impact of airflow obstruction on the survival of patients with esophageal carcinoma undergoing esophagectomy was performed.

**Results:**

Patients with airflow obstruction (102/821, 12.4%) had lower three-year overall (42/102, 58.8%) and progression-free survival rate (47/102, 53.9%) than those without airflow obstruction (*P *< 0.001). Multivariate analyses showed that airflow obstruction was an independent risk factor for overall survival (Hazard Ratio = 1.66; 95% CI: 1.17–2.35, *P *= 0.004) and disease progression (Hazard Ratio = 1.51; 95% CI: 1.1–2.08; *P = *0.01). A subgroup analysis revealed that the above results were more significant in male patients, BMI < 23 kg/m^2^ patients or late-stage cancer (stage III-IVA) (*P *= 0.001) patients and those undergoing open esophagectomy (*P *< 0.001).

**Conclusion:**

Preoperative airflow obstruction defined by FEV_1_/FVC ratio below LLN was an independent risk factor for mortality in esophageal cancer patients after trans-thoracic esophagectomy. Comprehensive management of airflow obstruction and more personalized surgical decision-making are necessary to improve survival outcomes in esophageal cancer patients.

## Introduction

Esophageal cancer (EC) is a highly aggressive malignancy with an inferior prognosis of 5-year survival rate of about 20% over the past decade worldwide (1). The incidence and healthcare burden of esophageal cancer in Eastern Asia were higher than in the rest of the world over the past decades (2). Though esophagectomy is an essential treatment for esophageal cancer, it is associated with a high incidence of postoperative complications (3, 4), and overall outcomes are still poor for late-stage esophageal cancer, especially in squamous cell cancer (5, 6).

Lung function is a criterion for eligibility for radical esophagectomy (7). Esophageal cancer patients undergoing esophagectomy should have good or at least not poor lung function, as many patients with severe chronic pulmonary disease are unsuitable for thoracic surgery. It is widely accepted that smoking is one of the relevant risk factors for esophageal cancer and chronic obstructive airway disease (8). Previous research demonstrated a high degree of overlap (7.1%–25%) of operable esophageal cancer patients with chronic obstructive pulmonary diseases (COPD) or asthma (9–11). Furthermore, chronic airway obstruction is directly related to the morbidity of esophagectomy, particularly concerning pulmonary complications and anastomotic leaks (10, 11). However, studies on the outcomes of patients with esophageal cancer and COPD or asthma were limited to postoperative morbidity rather than survival status. Though preoperative low vital capacity decreased the survival rate after radical esophagectomy for cancer (12), the impact of preoperative airway obstruction on long-term survival is unclear. Thus, an accurate assessment of the risk of airway obstruction in esophageal cancer patients is essential. We conducted this study to investigate the impact of preoperative airway obstruction on survival outcomes in patients with esophageal cancer after trans-thoracic esophagectomy. These findings shed light on patients’ long-term airway management after esophageal cancer surgery.

## Materials and methods

### Population

**Figure 1:**
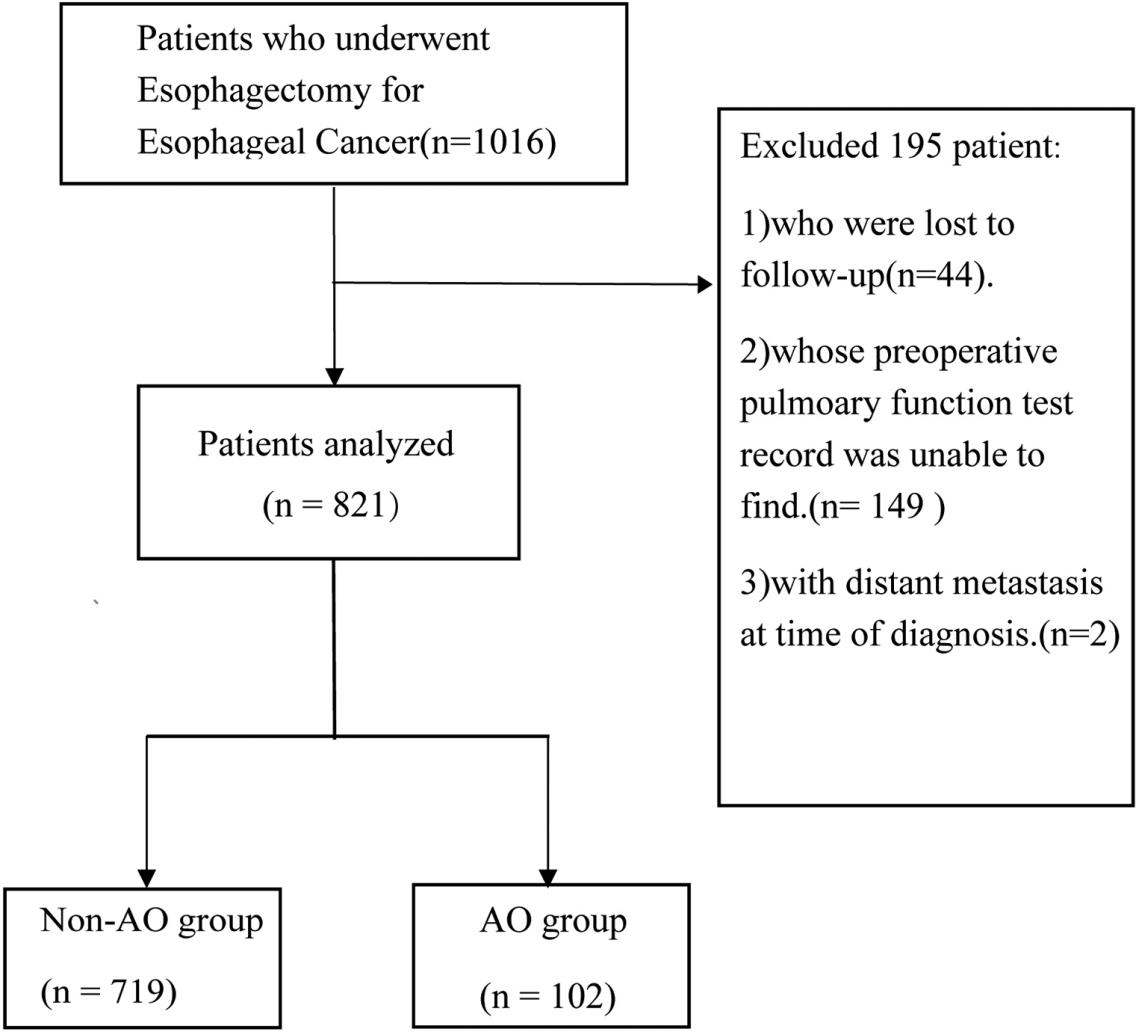
Study profile. Schematic diagram showing the study profile. The AFO group included patients with an FEV_1_/FVC ratio below the LLN. AFO: airflow obstruction.

This is a single-center, retrospective cohort study. From June 2012 to December 2015, 1,016 Chinese patients with esophageal cancer admitted to Zhongshan Hospitals, Fudan University (Shanghai, China), were evaluated and enrolled in the present study. All patients underwent radical trans-thoracic esophagectomy (Ivor-Lewis or McKeown procedure) with gastroesophageal reconstruction. Forty-four patients lost to follow-up, 149 patients without retrieved preoperative spirometry records, and two patients with distant metastasis at the time of diagnosis (M1) were excluded from the sample (Figure 1). This study was approved by the Ethics Committee of Zhongshan Hospital, Fudan University.

### Data collection for baseline patients' characteristics

Esophageal carcinoma and the stage were pathologically determined.

Information about patient characteristics and short-term postoperative complications before hospital discharge was obtained from the patient's medical records. Demographic characteristics, clinicopathological features, pulmonary function, and details of postoperative complications were collected and summarized in Tables 1, 2.

**Table 1:** Baseline demographics, clinicopathological and spirometric characteristics of Non-AFO and AFO patients.

	Total, *n* = 821	Non-airflow obstruction, *n* = 719	Airflow obstruction, *n* = 102	*p* value
Age, year^#^	61.2 (38–84)	60.9 (38–84)	62.5 (44–77)	0.04*
Gender				<0.001*
Male	626 (76.2)	525 (73)	91 (89.2)	
Female	195 (23.8)	194 (27)	11 (10.8)	
BMI, kg/m^2^	22.8 (14.8–34.5)	22.9 (14.8–34.5)	22.2 (15.6–30.5)	0.019*
Smoke				0.003*
Current or ever	355 (43.3)	297 (41.3)	58 (56.9)	
Never	466 (56.7)	422 (58.7)	44 (43.1)	
Pulmonary Function				
FEV_1_/FVC	77.51 (45.39–99.08)	79.57 (67.33–99.08)	63.01 (45.39–69.7)	<0.001*
FEV_1_, L	2.58 (0.97–4.53)	2.66 (1.08–4.53)	2.06 (0.97–3.64)	<0.001*
FVC, L	3.34 (1.09–5.74)	3.35 (1.09–5.74)	3.26 (1.54–5.22)	0.26
%FVC	92.93 (27.81–132.36)	93.55 (27.81–145.9)	88.52 (51.2–132.3)	<0.001*
DLCO^1^, mL/min mHg^−1^	19.33 (1.42–32.67)	19.58 (2.83–35.72)	17.4 (1.42–32.67)	0.002*
%DLCO^1^	91.51 (13.19–175/96)	92.93 (13.19–175.96)	80.95 (16.51–147.95)	<0.001*
Neoadjuvant Chemotherapy				0.39
Yes	84 (10.2)	78 (10.6)	8 (7.8)	
No	737 (89.8)	643 (89.4)	94 (92.2)	
Approach				0.72
Open	432 (52.6)	380 (52.9)	52 (50.98)	
MIE	389 (47.4)	339 (47.1)	50 (49.02)	
pG				0.47
G3	280 (34.1)	242 (33.7)	38 (37.4)	
G1-2	541 (65.9)	477 (66.3)	64 (62.6)	
pT				0.20
T0-1	278 (33.9)	250 (34.8)	28 (27.5)	
T2	206 (25.1)	178 (24.8)	28 (27.5)	
T3-4	337 (41)	291 (40.4)	46 (45)	
pN				0.35
N0	543 (66.1)	483 (67.2)	60 (58.9)	
N1	174 (21.2)	146 (20.3)	28 (27.4)	
N2-3	104 (12.7)	90 (12.5)	14 (13.7)	
Histology				0.018*
SCC	768 (93.5)	667 (92.8)	101 (99.0)	
Others	53 (6.5)	52 (7.2)	1 (1.0)	
Tumor length (cm)^#^	3.19 (1–10)	3.18 (1–10)	3.25 (1–8)	0.61
Tumor location				0.76
Upper	73 (8.9)	62 (8.6)	11 (10.8)	
Middle	486 (59.2)	426 (59.3)	60 (58.8)	
Lower	262 (31.9)	231 (32.2)	31 (30.4)	
PNI				0.39
Yes	129 (15.8)	110 (15.3)	19 (18.6)	
No	691 (84.2)	608 (84.6)	83 (81.4)	
LVSI				0.82
Yes	99 (12.1)	86 (12)	13 (12.7)	
No	722 (87.9)	633 (88)	89 (87.3)	

BMI, body mass index; FEV1/FVC, forced expiratory volume in 1s/vital capacity; FEV1, forced expiratory volume in 1s; FVC, forced vital capacity; %VC, %forced vital capacity; DLCO, diffusing capacity; MIE, Minimally invasive esophagectomy; pT, pathological T factor; pN, pathological N factor; pStage, pathological Stage. SCC, Squamous cell carcinoma; PNI, perineural invasion; LVSI, lymph-vascular space invasion.

* *p* value < 0.05.

^#^ Data are shown as median (range). All other data are shown as numbers (%) or mean (range).

^1^ Missing data. DLCO and % DLCO were missing for 3.3% (*N *= 27).

**Table 2:** Operative outcomes among the study populations.

		Airflow Obstrcution, *n* = 102	Non-airflow Obstruction, *n* = 719	Total, *n* = 821	*p* value
**Median hospital stay (days)** ^#^	**Preoperative**	3 (1–13)	3 (1–21)	3 (1–21)	0.42
	**Postoperative**	13 (7–105)	12 (6–197)	12 (6–197)	0.11
	**Total**	18 (9–108)	16 (8–200)	16 (8-200)	0.18
** Overall complications (**≥**Grade II)**		50 (49)	287 (39.9)	337 (41)	0.08
** Anastomotic leakage**		28 (27.5)	105 (14.6)	133 (16.2)	0.001*
** Pulmonary complications**		15 (14.7)	129 (17.9)	144 (17.5)	0.42
** Lung metastasis**		18 (17.65)	58 (8.1)	76 (8.9)	<0.001*
** Mediastinal lymph node metastasis**		10 (9.8)	95 (13.2)	105 (12.8)	0.34

* *p* value < 0.05.

^#^ Data are shown as median (range). All other data are shown as numbers (%).

### Evaluation of preoperative pulmonary function variables by spirometry

Spirometry was performed in Zhongshan hospital according to the ATS standards (13). Airflow obstruction was defined as a forced expiration volume in the first second (FEV1)/forced vital capacity (FVC) ratio was below the lower fifth percentile of a large healthy Chinese reference group (lower limit of normal, LLN) (14–16). The lower limit of normal (LLN) of FEV1/FVC was calculated with the formula in Supplementary Table S1. A website was developed by our team for convenient calculation and diagnosis of airflow obstruction (https://drpulmonary.shinyapps.io/AOdiagnosistool/). Given that FEV1/FVC decreases with increased age and most of the study population were over 50 years old, LLN definition of airflow obstruction was used to minimize false positives.

### Postoperative complications

Postoperative complications, including pulmonary complications (e.g., pneumonia, acute respiratory distress syndrome, and aspiration), anastomotic leakage, surgical site infection, cardiac complications, chyle leakage, thromboembolic events, recurrent laryngeal nerve paresis, and other complications were summarized. The severity of postoperative complications was classified according to the Clavien-Dindo classification as instructed by the International Consensus on Standardization of Data Collection for Complications Associated With Esophagectomy (17). Overall complications were defined as grade II and higher according to the Clavien-Dindo classification.

### Follow-Up and definition of recurrence

In principle, patients were reviewed through in-clinic follow-ups every three months in the first year and every six months after that for at least 3 years. Computed tomography of the neck, chest, and abdomen was examined every six months. Disease progression was defined as local recurrence of primary esophageal cancer, distant metastasis, or death due to any cause.

### Statistical analysis

All collected data were manually checked for completeness and consistency, and the continuous variables were tested for normality using the Shapiro–Wilk test. Normally distributed variables were compared using the *t*-test, and non-normally distributed ones were compared using the Mann–Whitney *U* test between airflow obstruction and non-airflow obstruction groups. Comparisons between the proportions were made using the *χ*2 test or Fisher's exact test. Survival was calculated using Kaplan–Meier survival curves and compared using the log-rank test. *P *< 0.05 was considered significant. Median follow-up time was calculated using the reverse Kaplan–Meier method (18). The Cox proportional hazards model was used for the univariate and multivariate analyses to identify independent risk factors associated with survival. Risk-adjusted, restricted *cubic* splines with 4 knots were used to model the possible non-linearity of the association between BMI and the risk of all-cause death (19, 20). The R *Code* for restricted cubic splines analysis is available on the GitHub repository: https://github.com/longerham/RCS#rcs. Data analysis was performed using R Foundation Statistical software (R 3.2.2) with ggplot2, forest plot, and survival packages (The R Foundation for Statistical Computing, Vienna, Austria).

## Results

### Distribution of characteristics in the study population

Among included 821 patients with esophageal cancer, 102 patients were with airflow obstruction (FEV1/FVC < LLN, AFO group), and the remaining 719 patients were classified as non-airflow obstruction (FEV_1_/FVC ≥ LLN, non-AFO group) patients. Table 1 showed that non-airflow obstruction patients were younger than airflow obstruction patients (mean 60.9 vs. 62.5 years; *P *< 0.001). Airflow obstruction was associated with male (*P *< 0.001), lower BMI (mean 22.2 vs. 22.9 kg/m^2^; *P *= 0.019), smoking history (*P *= 0.003), and squamous cell carcinoma (*P *= 0.018) No signiﬁcant differences in tumor grades (G), pathological T factor (pT); pathological N factor (pN), perineural invasion (PNI), lymph-vascular space invasion (LVSI), tumor length or tumor locations between two groups were discovered. Table 1 also demonstrated the differences in spirometric variables and operative procedures between AFO and non-AFO groups. FEV_1_/FVC, FEV_1_, %VC predicted, and DLCO variables in AFO group were significantly lower than those in non-AFO group.

### Short-term outcomes in AFO and non-AFO groups

Length of hospital stay and incidence of overall complications, pulmonary complications, and anastomotic leaks were given in Table 2. Airflow obstruction patients showed significantly higher rate of anastomotic leakage than non-airflow obstruction patients (27.5% vs. 14.6%; *P *< 0.001). However, there were no significant differences between the groups in the length of hospital stay and rates of pulmonary complications.

### Impact of airflow obstruction on survival of esophageal cancer patients

**Figure 2:**
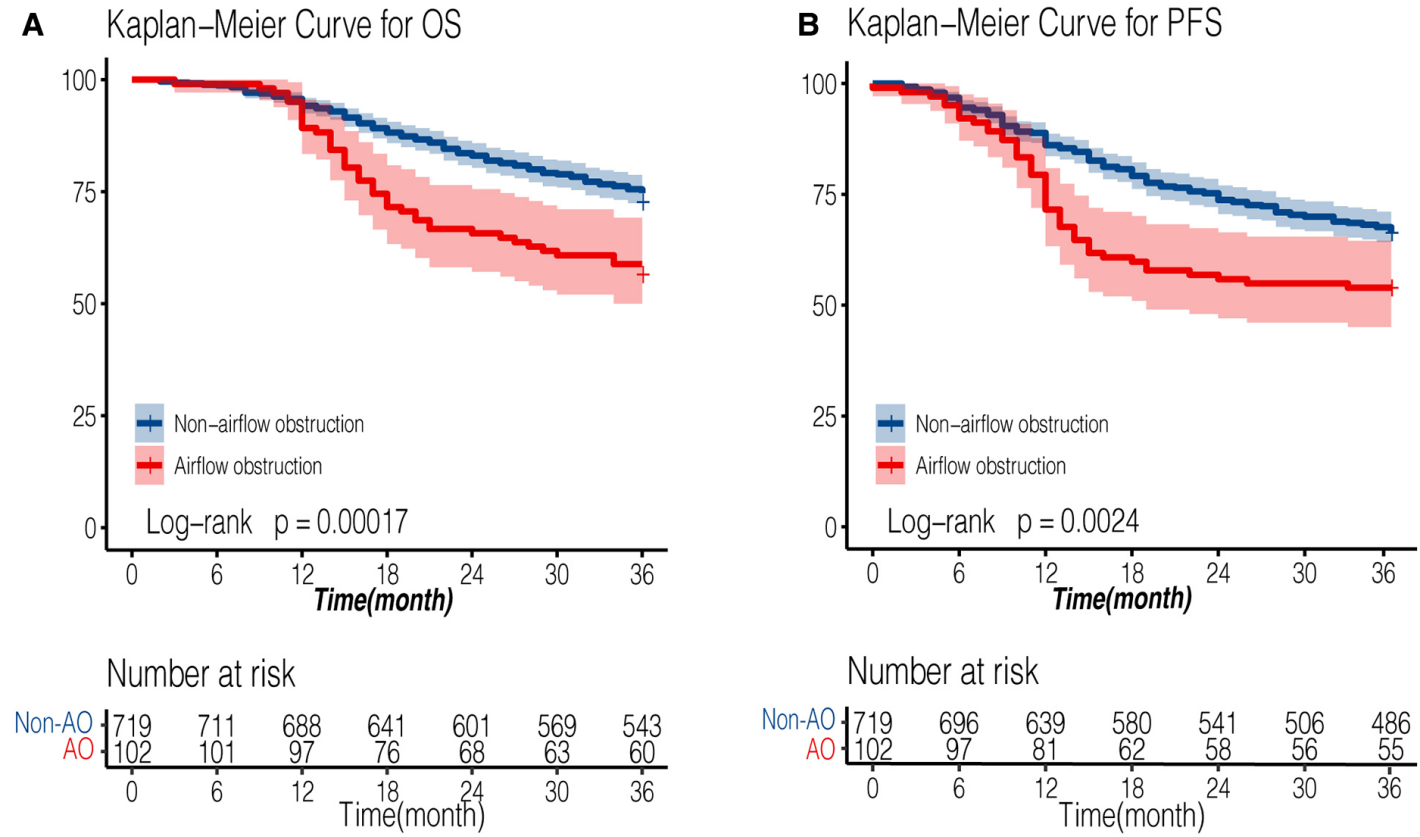
Kaplan-Meier survival curve of AFO and non-AFO groups in patients undergoing esophagectomy for esophageal cancers. (**A**): overall survival curve; (**B**): progression-free survival curve). AFO: airflow obstruction.

The median follow-up time was 54 months for all patients, while the median follow-up time was 53.6 months (95% CI: 51.9–56.1) in non-AFO group and 55.9 months (95% CI: 52.2–59.1) in AFO group (*P *= 0.61). The 3-year overall survival (OS) rates were 75.5% and 58.82%, and 3-year progression-free survival (PFS) rates were 67.5% and 53.92% in non-airflow obstruction and airflow obstruction groups, respectively. The airflow obstruction patients' OS and PFS rates were significantly worse than those of non-obstruction patients (*P *< 0.001 and *P *= 0.002, respectively, Figure 2). Table 3 presents a multivariate Cox regression analysis performed on factors showing significance in the univariate analysis (age, gender, smoking status, surgical approach, pT, pN, G, PNI, LVSI, and anastomotic leakages). Airflow obstruction turned out to be an independent risk factor for OS (Hazard Ratio = 1.66; 95%CI: 1.17–2.35, *P *= 0.004) and PFS (Hazard Ratio = 1.51; 95% CI: 1.1–2.08; *P *= 0.01) in esophageal cancer patients.

**Table 3:** Cox proportional hazards regression models for predictors of overall survival (OS) and progression-free survival (PFS).

Characteristics	No.	*Univariate analysis*		*Multivariate analysis*	
		HR (95% CI)	*p* value	HR (95% CI)	*p* value
***Overall survival***					
Airflow Obstruction	102	1.89 (1.35–2.64)	<0.001*	1.66 (1.17–2.35)	0.004*
Age >70 (vs. ≤70)	83	1.5 (1.03–2.2)	0.035*	1.27 (0.86–1.88)	0.23
Male	626	2.33 (1.59–3.45)	<0.001*	1.72 (1.12–2.63)	0.013*
BMI ≥23 (vs. <23)	402	1.12 (0.69–1.16)	0.39		
Smoker	355	1.31 (1.01–1.7)	0.046*	1.06 (0.8–1.41)	0.69
MIE approach (vs. Open approach)	389	0.62 (0.47–0.81)	<0.001*	0.69 (0.53–0.92)	0.011*
Complications	337	1.14 (0.88–1.49)	0.32		
Anastomotic leakage	133	1.47 (1.07–2.03)	0.019*	1.34 (0.96–1.86)	0.09
Neoadjuvant chemotherapy	86	1.22 (0.81–1.83)	0.35		
G3 (vs. G1-2)	280	1.78 (1.37–2.32)	<0.001*	1.25 (0.95–1.65)	0.11
PNI	129	2.04 (1.51–2.77)	<0.001*	1.1 (0.78–1.54)	0.59
LVSI	99	2.39 (1.74–3.3)	<0.001*	1.62 (1.14–2.29)	0.007*
pT					
T0-1	278	REF	REF	REF	REF
T2	206	2.22 (1.45–3.41)	<0.001*	1.5 (0.96–2.35)	0.077
T3-4	337	3.87 (2.67–5.61)	<0.001*	2.21 (1.47–3.29)	<0.001*
pN					
N0	543	REF		REF	
N1	174	2.32 (1.69–3.19)	<0.001*	1.77 (1.27–2.45)	<0.001*
N2-3	104	4.62 (3.36–6.34)	<0.001*	2.64 (1.86–3.76)	<0.001*
***Progression-free survival***					
Airflow Obstruction	102	1.62 (1.18–2.21)	0.003*	1.51 (1.1–2.08)	0.011*
Age >70 (vs. ≤70)	83	1.34 (0.94–1.9)	0.102		
Male	626	1.78 (1.32–2.44)	<0.001*	1.39 (1.01–1.92)	0.043*
Smoker	355	1.23 (0.98–1.55)	0.076		
MIE approach (vs. Open approach)	389	0.66 (0.51–0.83)	0.001*	0.77 (0.60–0.98)	0.03*
Complications	337	1.14 (0.91–1.44)	0.26		
Anastomotic leakage	133	1.35 (1.01–1.8)	0.045*	1.23 (0.91–1.66)	0.17
Neoadjuvant chemotherapy	86	1.19 (0.83–1.71)	0.36		
G3 (vs. G1-2)	280	1.61 (1.27–2.03)	<0.001*	1.11 (0.87–1.42)	0.41
PNI	129	1.95 (1.48–2.56)	<0.001*	1.05 (0.78–1.42)	0.74
LVSI	99	1.95 (1.8–3.19)	<0.001*	1.563 (1.2–2.22)	0.002*
pT					
T0-1	278	REF		REF	
T2	206	1.96 (1.36–2.83)	<0.001*	1.48 (1.02–2.17)	0.041*
T3-4	337	3.74 (2.73–5.12)	<0.001*	2.43 (1.72–3.42)	<0.001*
pN					
N0	543	REF		REF	
N1	174	2.18 (1.65–2.87)	<0.001*	1.67 (1.25–2.22)	<0.001*
N2-3	104	4.05 (3.05–5.4)	<0.001*	2.44 (1.78–3.35)	<0.001*

HR, hazard ratio; CI, conﬁdence interval; MIE, minimally invasive esophagectomy; pT, pathological T factor; pN, pathological N factor; PNI, perineural invasion; LVSI, lymph-vascular space invasion.

* *P *< 0.05.

### Subgroup survival analysis

**Figure 3:**
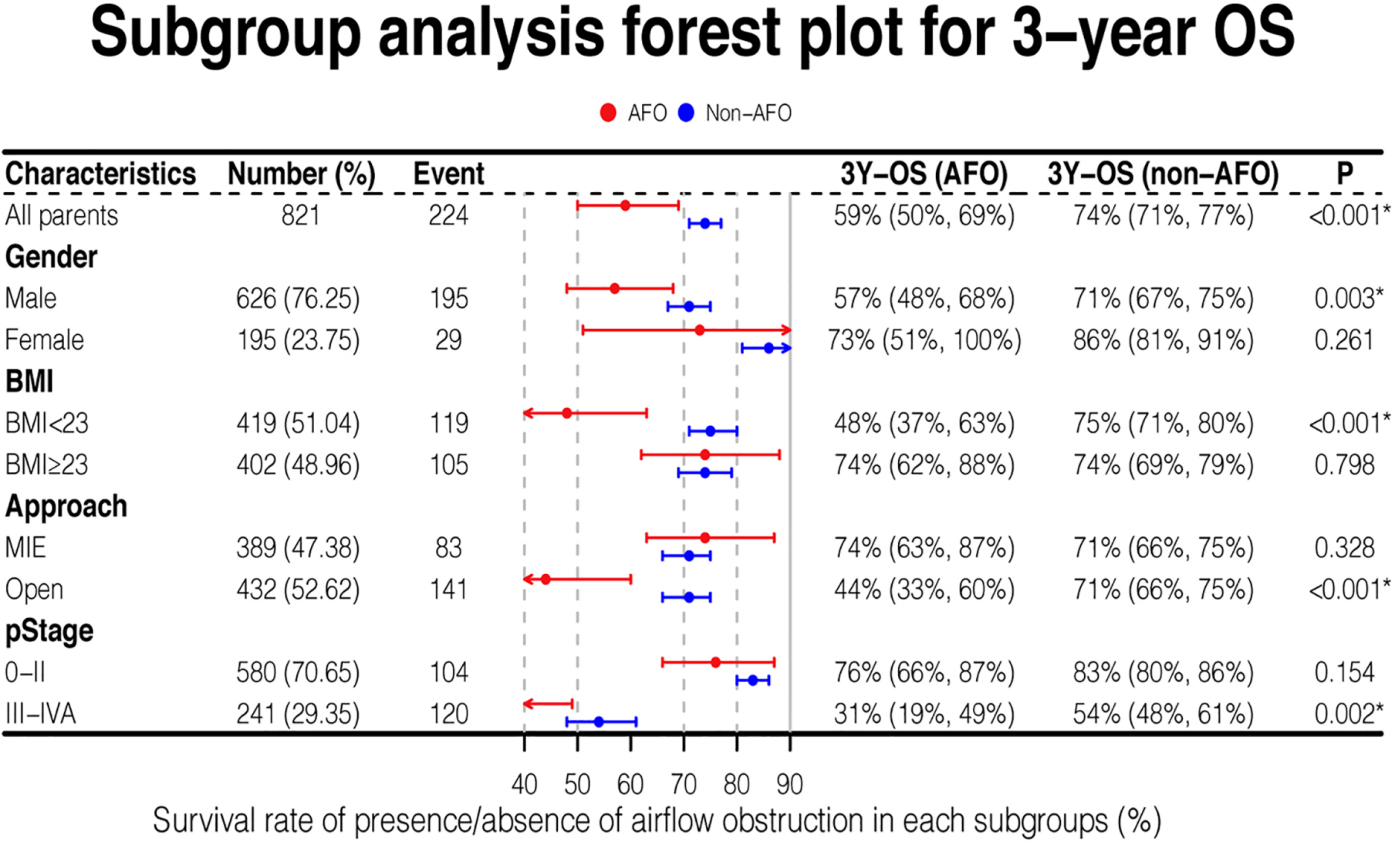
Forest plot for subgroups analysis of overall survival.

Overall survival stratified by several covariates was analyzed. When patients were male (*P *= 0.003), with BMI < 23 kg/m^2^ (*P *< 0.001), with late-stage cancer (stage III-IVA) (*P *= 0.002), or undergoing open esophagectomy (*P *< 0.001), the overall survival was significantly shorter in AFO group compared with non-AFO group. Other covariates showed no differences in survival between the two groups (Figure 3).

Notably, the 3-year survival rate of airflow obstruction with open surgical procedure or stage III-IVA was 44% and 31%, respectively, which were much lower than those in any other subgroups analyzed.

### Impact of airflow obstruction with BMI < 23 kg/m^2^ on survival of esophageal cancer patients

**Figure 4:**
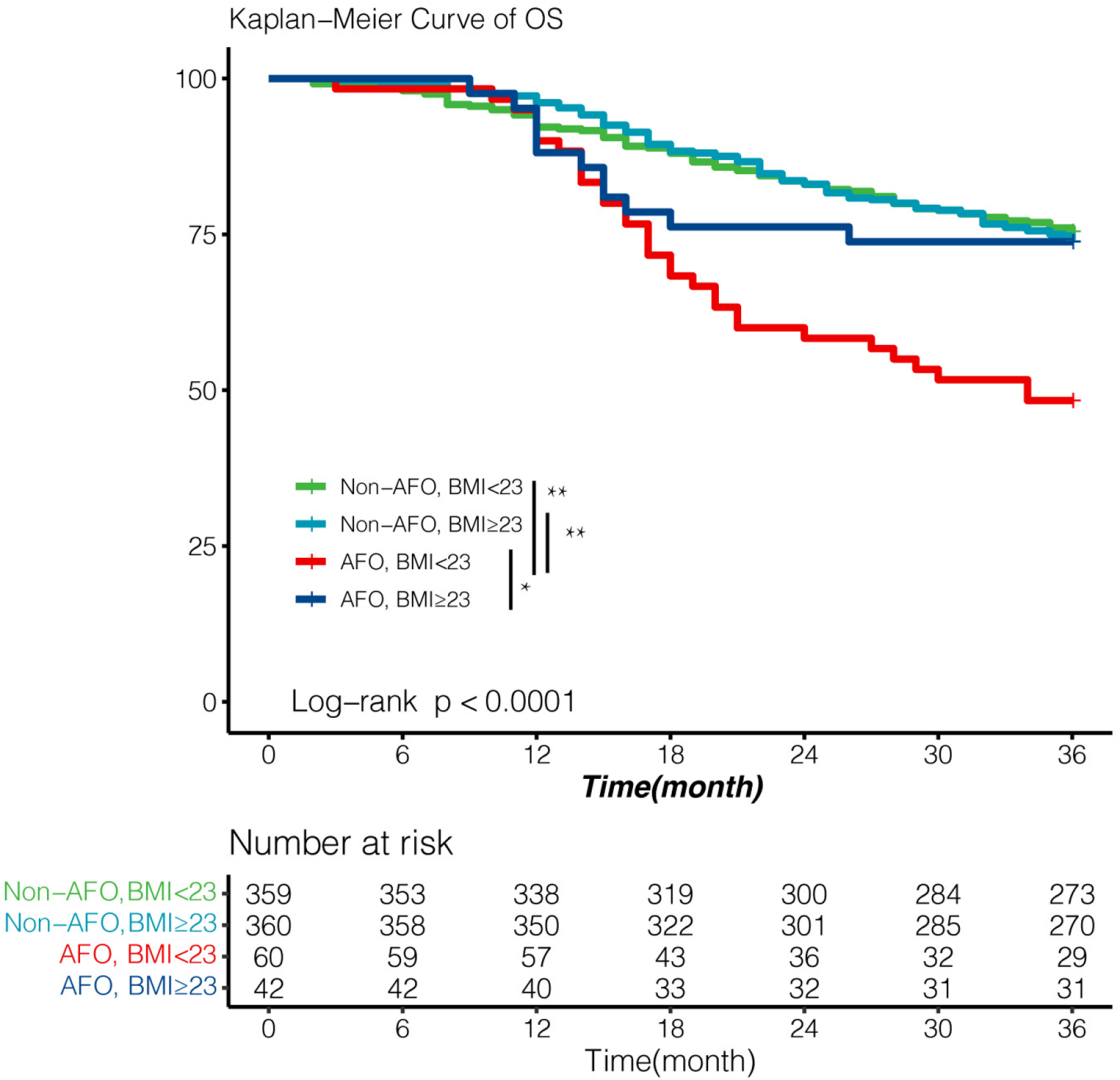
Survival according to airflow obstruction and BMI with cut-off value 23 kg/m^2^. Patients with both airflow obstruction and BMI < 23 showed significantly poor OS (3-year OS; 48%). Survival outcomes of other three patients were essentially equivalent (3-year OS; 75%, 74.0%, 74% respectively). Survival was analyzed by Kaplan–Meier method using the log-rank test. AFO: airflow obstruction. BMI:body mass index. *p value < 0.05, **p value < 0.01.

Among all baseline variables, BMI was significantly lower in the obstruction group than in non-airflow obstruction group (22.2 vs. 22.9, *P *= 0.019). We evaluated the comprehensive impact of airflow obstruction and BMI on survival. A BMI of 23 kg/m^2^ is used to distinguish whether a patient is overweight. Patients with both BMI < 23 kg/m^2^ and airflow obstruction showed inferior outcomes (3-year OS: 48%, Figure 3), which was significantly worse than that of patients in the other three groups (all *P *< 0.05, Figure 4). However, the BMI value was not related to the overall survival of the entire study population (Table 3).

To further validate this finding, we performed a univariate Cox regression analysis in AFO group (Supplementary Table S2). Variables with *P *< 0.05 in the univariate Cox regression analysis were included in the multivariate Cox proportional splines model to reflect the non-linear relation between all-cause mortality and BMI as a continuous variable. Hazard ratios of mortality decreased more as BMI increased (Supplementary Figure S1B) in airflow obstruction patients, compared with that in the whole population (Supplementary Figure S1A).

### Airflow obstruction promotes lung metastasis in esophageal cancer patients

It was noteworthy that lung metastasis was associated with airflow obstruction (Table 2, *P *= 0.01). The evaluation of risk factors for lung metastasis in esophageal cancer patients is shown in Table 4. In multivariate Cox regression analysis, airflow obstruction was associated with a significantly increased probability (Hazard Ratio = 2.22; 95% CI: 1.31–3.78; *P* = 0.003) of lung metastasis from the primary tumor. The risk for lung metastasis also significantly increased when the pathological N factor was larger than 0 (Hazard Ration = 1.73; 95% CI: 1.07–2.78; *P *= 0.024).

**Table 4:** Univariate and multivariate Cox proportional hazards regression analysis for the evaluation of risk factors for lung metastasis within 3 years after esophagectomy.

	Events	Univariate HR (95% CI)	*P* value	Multivariate HR (95% CI)	*P* value
Airflow Obstruction	18	2.40 (1.4–4)	<0.001*	2.22 (1.31–3.78)	0.005*
Male	60	1.2 (0.67–2.08)	0.52		
MIE approach (vs. Open approach)	31	0.77 (0.48–1.19)	0.23		
G3 (vs. G1-2)	29	1.21 (0.75–1.9)	0.45		
pT3-4 (vs. pT0-2)	41	1.7 (1.1–2.7)	0.02*	1.4 (0.86–2.3)	0.17
pN1-3 (vs. pN0)	38	2 (1.3–3.2)	<0.001*	1.73 (1.07–2.78)	0.024*
PNI	17	1.61 (0.91–2.77)	0.09	1.19 (0.67–2.11)	0.54
LVSI	12	1.39 (0.77–2.56)	0.29		
Anastomotic leakage	16	1.4 (0.8–2.4)	0.25		
Pulmonary complications	17	1.4 (0.82–2.4)	0.22		

HR, hazard ratio; CI, conﬁdence interval; MIE, minimally invasive esophagectomy; pT, pathological T factor; pN, pathological N factor; PNI, perineural invasion; LVSI, lymph-vascular space invasion.

* *P *< 0.05.

## Discussion

This is a single-center-based retrospective cohort study of patients with esophageal cancer. And it is the first study on the prognosis impact of preoperative airflow obstruction defined as FEV_1_/FVC < LLN for esophageal cancer. Our findings suggest that (i) airflow obstruction was observed in 12.4% of patients receiving esophageal cancer surgery, (ii) preoperative airflow obstruction was an independent prognostic factor for 3-year OS and PFS following trans-thoracic esophagectomy. (iii) preoperative airflow obstruction was an independent risk factor for pulmonary metastasis in esophageal cancer.

The impact of airway obstruction on patients' survival outcomes should not be surprising. Trans-thoracic esophagectomy affects the activity of the chest wall and the lung. Meanwhile, the stomach moves upward and squeezes into the lungs after esophagogastrostomy, resulting in limited pulmonary dilatation and accelerated lung function decline. Patients with chronic airflow obstruction diseases (COPD and asthma, for instance) may be more susceptible to anastomotic leakages and infections, which detrimentally affect survival by delaying recovery or leading to death (21, 22).

Subgroup analysis shed light on the most sensitive population to airflow obstruction. Minimally invasive esophagectomy (MIE) could reduce the response of the organism, accelerate recovery and maintain postoperative pulmonary function (23, 24). Patients with airflow obstruction may particularly benefit from MIE. Moreover, airflow obstruction worsened survival of stage III-IVA esophageal cancer; but showed no difference in patients with stage 0-II cancer. This is probably because late-stage cancer patients have deteriorating disease manifestations and declinin**g** quality of life (25, 26). The presence of airflow obstruction worsens the cognitive and overall status at certain levels (27, 28), playing an adjunctive role in the lethal effects of EC. But in the early stages, the follow-up was relatively short, and most of them did not experience the outcome event. The sex difference might be because insufficient female patients led to investigation bias.

A previous study demonstrated that patients with lower BMI had a faster FEV_1_/FVC decline and more symptoms than patients with higher BMI (29). In line with these prior results (30, 31), patients with airflow obstruction in our study had lower BMI. It is noteworthy that patients with airflow obstruction but BMI ≥23 kg/m^2^ exhibited as good survival outcomes as the non-airflow obstruction group, which suggested higher BMI could be protective in esophageal cancer patients complicated with airflow obstruction. Therefore, we assume that BMI or overall nutrition status could partly explain our findings on survival outcomes.

Another interesting phenomenon was that airway obstruction facilitated the lung spread of esophageal cancer. This finding echoes the impact of smoking (32) since smoking is highly correlated to airflow obstruction. The “seed-and-soil hypothesis” partially explains this finding (33, 34). Airway obstruction usually coexists with the remodeling of the airway epithelium and alterations of the distribution of inflammatory cells, providing an ideal micro-environment (soil) for tumor cells (seed) colonization and growth (35). Therefore, our findings shed new light on the mechanism of lung metastasis of esophageal cancer.

Unfortunately, in our study, only 25 (25/102, 24.5%) were diagnosed with chronic obstructive airway diseases before the esophagectomy. Almost all patients (95/102, 93.1%) were without sustained lung-directed therapy. Although undiagnosed airflow obstruction subjects appeared healthier than those with a diagnosis, their prognosis was worse than subjects without airflow obstruction^15^. Our work suggests that preoperative airflow obstruction and potential obstructive airway diseases should be given more attention. Perioperative and long-term airway intervention deserves further investigation to improve survival outcomes.

There are some limitations to our study. First, the median follow-up duration was 54 months in the whole study population, while more extended follow-up periods may provide detailed information on EC prognosis, especially in stage 0-II patients. Secondly, the sample size of patients receiving neoadjuvant therapy was not enough. The interaction between airflow obstruction and neoadjuvant treatment remains to be demonstrated. Finally, 93.5% of patients in our cohort were with esophageal squamous cell carcinoma, whose BMI was generally lower than average (36). It remains unclear whether our conclusions apply to western countries, where adenocarcinoma is the primary pathological type.

## Conclusion

Airflow obstruction is a common comorbidity in patients with esophageal cancer. Patients with airflow obstruction had more postoperative complications and shorter 3-year OS and PFS after trans-thoracic surgery for esophageal cancer. BMI or overall nutrition status could partly explain these effects. More attention is needed to manage airflow obstruction in esophageal cancer patients comprehensively. We should incorporate the patient's respiratory condition into the surgical decision-making process to reach a better prognosis.

## Data Availability

The raw data supporting the conclusions of this article will be made available by the authors, without undue reservation.
