# Supplementary material for: Concomitant preoperative airflow obstruction confers worse prognosis after trans-thoracic surgery for esophageal cancer
Source: Front Surg. 2023 Jan 16;9:966340. doi: 10.3389/fsurg.2022.966340 (PMC9885207; doi:10.3389/fsurg.2022.966340)
Supplement: Supplementary file 1 [file Datasheet1.docx]

Supplementary Material

# Supplementary Tables

# Supplementary Table 1. Reference equations of lower limit of normal(LLN) of FEV1/FVC for males and female.

| Male | Female |
| --- | --- |
| Mspline#=(-3)×10-7×Age3+7*10-5×Age2  -0.006×Age+0.1447 | Mspline=(-3)×10-7×Age3+7×10-5×Age2  -0.006×Age+0.1447 |
| Sspline = 4×10-5×Age2+0.0069×Age-0.384 | Sspline = (-2)×10-7×Age2+0.0014×Age-0.0567 |
|  | Lspline = 0.0002×Age2-0.0461×Age+1.6798 |
| M = exp[5.12465 - 0.11084 × ln(height in cm) - 0.03726 × ln(age in year) + Mspline] | M = exp[5.01251 - 0.07453 × ln(height in cm) - 0.05649 × ln(age in year) + Mspline] |
| S = exp[-8.3848 + 1.2030 × ln(height in cm) - 0.1214 × ln(age in year) + Sspline] | S = exp[-3.1856 + 0.1298 × ln(age in year) + Sspline] |
| L = 5.2356 - 0.9546 × ln(age in year) | L = 9.601 - 2.177 × ln(age in year) + Lspline |
| LLN (5th) = exp[ln(M) + ln(1-1.645×L×S)/L] | LLN (5th) = exp[ln(M) + ln(1- 1.645 × L × S)/L] |

#Spline is an age-specific contribution from the spline function.

# Supplementary Table 2. Univariate cox proportional hazards regression analysis for overall survival(OS) in AFO patients.

| Characteristics | ***Univariate analysis*** | |
| --- | --- | --- |
|  | HR(95%CI) | p value |
| Age | 0.94(0.9-.0.98) | 0.007* |
| Male | 0.52(0.16 - 1.67) | 0.27 |
| BMI | 0.91(0.83-1.01) | 0.077 |
| Open esophagectomy  (vs. MIE) | 2.55(1.32-4.92) | 0.005* |
| G3(vs. G1-2) | 1.84(1.01-3.38) | 0.048 |
| NAC | 1.4(0.5-3.93) | 0.522 |
| Complications | 01.33(0.72-2.44) | 0.35 |
| LVSI | 1.42(0.64-3.23) | 0.381 |
| PNI | 1.72(0.87-3.49) | 0.117 |
| pT |  |  |
| T0-1 | REF | REF |
| T2 | 4.33(1.21-15.51) | 0.025* |
| T3-4 | 7.7(2.34-25.36) | 0.001* |
| pN |  |  |
| N0 | REF | REF |
| N1 | 2.88(1.42-5.83) | 0.003* |
| N2-3 | 4.49(2.05-9.82) | <0.001* |

HR: Hazard Ratio; CI: conﬁdence interval. * p value< 0.05.

# Supplementary Figure


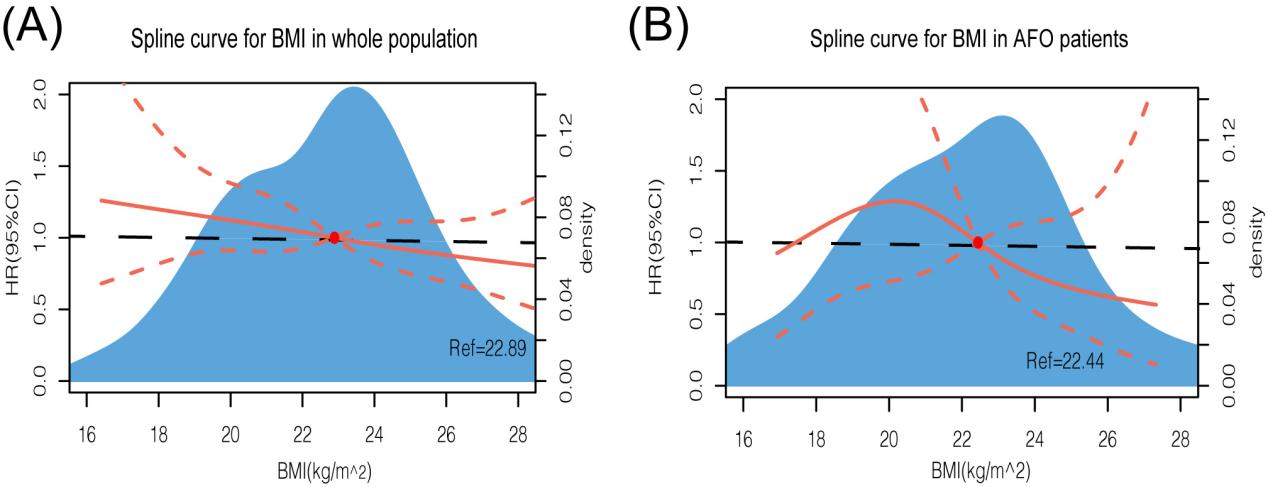


# Supplementary Figure 1. The association between BMI and the risk of overall death hazard ratios is shown for the association between BMI and the risk of overall mortality in the whole population(Fig.S1.a) and AFO patients(Fig.S1.b). The who people analyses were adjusted for AFO, sex, smoking status, surgical approach, anastomotic leakage, tumor grade, PNI, LVSI, pT and pN. The AFO group was adjusted for age, surgical approach, pT ,and pN. The dotted lines show 95% confidence intervals.
